# XFM-guided delivery of imaging-visible human mesenchymal stem cells into the pericardial space in a porcine model

**DOI:** 10.1186/1532-429X-14-S1-P63

**Published:** 2012-02-01

**Authors:** Yingli Fu, Nicole Azene, Tina Ehtiati, Aaron Flammang, Jens Guehring, Wesley Gilson, Judy A Cook, Clifford R Weiss, Peter V Johnston, Dara Kraitchman

**Affiliations:** 1 Radiology, Johns Hopkins University, Baltimore, MD, USA; 2 Molecular & Comparative Pathobiology, Johns Hopkins University, Baltimore, MD, USA; 3 Siemens Corporate Research, Baltimore, MD, USA; 4 Siemens Healthcare, Erlangen, Germany, USA; 5 Medicine, Johns Hopkins University, Baltimore, MD, USA

## Background

Transmyocardial delivery of stem cells in the setting of acute myocardial ischemia has shown promise to prevent adverse ventricular remodeling_1_. However, the efficacy is limited by poor cell retention and low survival rates, even for autologous cells. Intrapericardial delivery of microencapsulated stem cells may offer a local and minimally invasive route to enhance cell survival and retention. In this study, we assess the delivery of human mesenchymal stem cells (hMSCs) in a radiopaque microcapsule to the pericardium using x-ray fused with magnetic resonance imaging (XFM) in a porcine model.

## Methods

**Figure 1:**
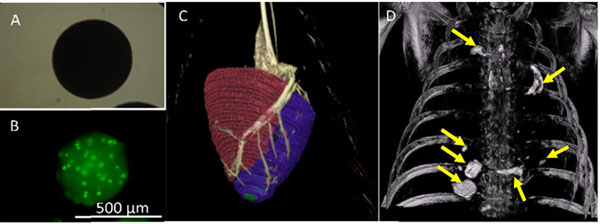
A) Microscopic view of BaCaps. B) Fluorescence image of Ba-encapsulated hMSCs demonstrating high cell viability (green) 2 days after encapsulation. C) XFM of the pig heart showing coronary vasculature (gray) and ventricular boundaries from MRI (colors). D) Cardiac-gated c-arm CT image showing the persistence of BaCaps in pericardial space one week post delivery (arrows).

Stem cell microencapsulation was performed using a modification of the alginate microencapsulation method by the addition of 10% (w/v) BaSO4 (BaCaps, Fig.1A)_2_. Female Yorkshire pigs (~25 kg, n=7) were randomized to receive either empty BaCaps (~10 ml), naked hMSCs (1x10_8_), saline, or BaCaps with hMSCs (8x10_7_) using a percutaneous approach. Prior to delivery, cine breath-held short-axis images were acquired to determine ventricular function (Argus, Siemens). A navigator-gated 3D whole heart MRI (1.5T Espree, Siemens SSFP, TR/TE=290/1.67 ms, FOV=320x240 mm, image matrix=256x173, iPAT=2, slice=64, slice thickness=2 mm) was also obtained to define ventricular borders for intrapericardial injection. A cardiac-gated c-arm CT (dynaCT, Axiom Artis dFA, Siemens, 190° rotation; 0.5° angle; 20s acquisition; 48 cm FOV) was then obtained and fused with the whole heart MRI and overlaid on live fluoroscopy to guide percutaneous access to the pericardial space. For chronic studies, MRI and c-arm CT imaging were repeated one week after injection. Animals were sacrificed immediately or one week post-delivery for histological analysis.

## Results

The viability of Ba-encapsulated hMSCs was 94.8±6% two days after encapsulation (Fig.1B). Using XFM (Fig.1C), successful puncture and delivery of BaCaps or Ba-encapsulated hMSCs was achieved in all animals. BaCaps were detected on fluoroscopic and c-arm CT images immediately and one week after delivery (Fig.1D). Whereas BaCaps were free floating immediately after delivery, at one week the BaCaps had consolidated as a pseudo epicardial tissue patch. Cardiac function was maintained 1 week post-delivery (LVEF: 36.8±4% at baseline vs. 41.5±5% at 1 week, n=5). Pericardial adhesions and effusion were absent at one week.

## Conclusions

XFM-guided intrapericardial injection of Ba-encapsulated hMSCs was safe and reliable. This approach holds promise for safe delivery of allogeneic cellular therapeutics to the heart in patients without pericardial effusion.

## Funding

Funding was provided by grants: R21/R33-HL89029, MD-SCRFII-0399 and Siemens healthcare.

